# A Rigidifying Salt-Bridge Favors the Activity of Thermophilic Enzyme at High Temperatures at the Expense of Low-Temperature Activity

**DOI:** 10.1371/journal.pbio.1001027

**Published:** 2011-03-15

**Authors:** Sonia Y. Lam, Rachel C. Y. Yeung, Tsz-Ha Yu, Kong-Hung Sze, Kam-Bo Wong

**Affiliations:** 1 School of Life Sciences, Centre for Protein Science and Crystallography, The Chinese University of Hong Kong, Shatin, Hong Kong SAR, China; 2 Department of Chemistry, University of Hong Kong, Hong Kong SAR, China; Brandeis University, United States of America

## Abstract

**Background:**

Thermophilic enzymes are often less active than their mesophilic homologues at low temperatures. One hypothesis to explain this observation is that the extra stabilizing interactions increase the rigidity of thermophilic enzymes and hence reduce their activity. Here we employed a thermophilic acylphosphatase from *Pyrococcus horikoshii* and its homologous mesophilic acylphosphatase from human as a model to study how local rigidity of an active-site residue affects the enzymatic activity.

**Methods and Findings:**

Acylphosphatases have a unique structural feature that its conserved active-site arginine residue forms a salt-bridge with the C-terminal carboxyl group only in thermophilic acylphosphatases, but not in mesophilic acylphosphatases. We perturbed the local rigidity of this active-site residue by removing the salt-bridge in the thermophilic acylphosphatase and by introducing the salt-bridge in the mesophilic homologue. The mutagenesis design was confirmed by x-ray crystallography. Removing the salt-bridge in the thermophilic enzyme lowered the activation energy that decreased the activation enthalpy and entropy. Conversely, the introduction of the salt-bridge to the mesophilic homologue increased the activation energy and resulted in increases in both activation enthalpy and entropy. Revealed by molecular dynamics simulations, the unrestrained arginine residue can populate more rotamer conformations, and the loss of this conformational freedom upon the formation of transition state justified the observed reduction in activation entropy.

**Conclusions:**

Our results support the conclusion that restricting the active-site flexibility entropically favors the enzymatic activity at high temperatures. However, the accompanying enthalpy-entropy compensation leads to a stronger temperature-dependency of the enzymatic activity, which explains the less active nature of the thermophilic enzymes at low temperatures.

## Author Summary

Although enzymes from thermophiles thriving in hot habitats are more stable than their mesophilic homologs, they are often less active at low temperatures. One theory suggests that extra stabilizing interactions found in thermophilic enzymes may increase their rigidity and decrease enzymatic activity at lower temperatures. We used acylphosphatase as a model to study how flexibility affects enzymatic activity. This enzyme has a unique structural feature in that an invariant arginine residue, which takes part in catalysis, is restrained by a salt-bridge in the thermophilic homologs but not in its mesophilic homologs. Here, we demonstrate the trade-offs between flexibility and enzymatic activity by disrupting the salt-bridge in a thermophilic acylphosphatase and introducing it in the mesophilic human homolog. Our results suggest that the salt-bridge is a structural adaptation for thermophilic acylphosphatases as it entropically favors enzymatic activity at high temperatures by restricting the flexibility of the active-site residue. However, at low temperatures the salt-bridge reduces the enzymatic activity because of a steeper temperature-dependency of activity.

## Introduction

To cope with the extremely hot habitats, thermophilic enzymes isolated from organisms thriving in these environments have usually evolved towards high thermal stability. Although thermophilic and mesophilic enzymes are comparably active at their respective temperatures where these enzymes function, thermophilic enzymes are often less active at lower temperatures [1],[2]. To date, this reduced activity of the thermophilic enzymes at low temperatures remains only partially understood. Comparative study of activity, stability, and flexibility relationships of homologous enzymes from thermophilic, mesophilic, and psychrophilic enzymes showed that enzyme flexibility is often correlated with its activity but is inversely related to its stability [1]–[9]. Thermophilic enzymes generally acquire larger values of activation entropy (ΔS^#^) and enthalpy (ΔH^#^) than those of their mesophilic and psychrophilic homologues [3],[10]. One popular interpretation of this observation is that the extra stabilizing interactions found in thermophilic enzymes will result in a more rigid enzyme. Increased rigidity has been proposed to explain why thermophilic enzymes are less active at low temperatures, while optimizing local flexibility is a structural adaptation of psychrophilic enzymes to remain active at low temperatures [1],[2],[11].

**Figure 1:**
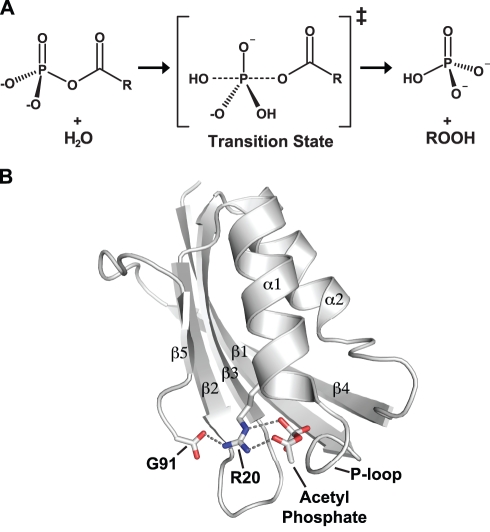
Acylphosphatases use an invariant arginine residue to catalyze the hydrolysis of its substrates. (A) The transition state of the enzyme-catalyzed hydrolysis of acylphosphate. (B) Schematic representation of the thermophilic PhAcP. The substrate acylphosphate was modeled to the active-site cradle, P-loop, by docking and molecular modeling [14]. The role of the active-site arginine residue (Arg-20) is to stabilize the negative charges in the transition state. In the structures of all thermophilic acylphosphatases determined to date, the active-site arginine residue forms a salt-bridge with the C-terminal carboxyl group.

Here we used a pair of thermophilic/mesophilic acylphosphatases homologues (acylphosphatase from hyperthermophilic archaeon *Pyrococcus horikoshii* (PhAcP) and human common-type acylphosphatase (HuAcP)) as a model to study how local flexibility of the active site affects the enzymatic activity. Acylphosphatases catalyze the hydrolysis of acylphosphates to phosphates and carboxylates, by providing an invariant arginine residue (Arg20 in PhAcP, Arg23 in HuAcP) that stabilizes the negative charges in the transition state (Figure 1) [12]–[14]. Despite remarkable conservation of sequence and structure at the active site, the catalytic activity of the thermophilic PhAcP is significantly poorer as compared with its mesophilic homologue HuAcP at low temperatures [14]. To date, the crystal structures of three thermophilic acylphosphatases and four mesophilic acylphosphatases are available (Figure S1). Structural comparison of the crystal structures reveals that this active-site residue Arg-20 forms a salt-bridge with the C-terminal carboxyl group of the glycine residue Gly-91 in PhAcP (Figure 1B) [14]. This salt-bridge is also present in thermophilic acylphosphatases from *Sulfolobus solfataricus* (PDB: 2bjd) [15] and *Thermus thermophilus* (PDB: 1ulr), but not in mesophilic acylphosphatases from human [16], cattle [13], Drosophila [17], and *Bacillus subtilis* (PDB: 2vh7, 2acy, 1urr, 3br8) (Figure S1). In PhAcP, the formation of the salt-bridge is facilitated by the C-terminal glycine, which can adopt an unusual φ angle of ∼180°. In the cases of thermophilic acylphosphatases from *T. thermophlius* and *S. solfataricus*, the C-terminal carboxylate groups are brought in the position to form the salt-bridge by having one less residue. The presence of the salt-bridge probably increases the rigidity of the active-site residue in thermophilic PhAcP by locking the guanido group of Arg-20 to prevent conformational fluctuation during catalysis.

In this study, we have shown that this salt-bridge is largely responsible for the observed differences in activation energy, and hence the temperature-dependency of enzymatic activity between thermophilic and mesophilic acylphosphatases. By disrupting the salt-bridge, the activation energy for the thermophilic enzyme was converted to mesophilic-like. Parallel findings were obtained by introducing the salt-bridge into the mesophilic HuAcP. Analysis of thermodynamics parameters showed that the removal of the salt-bridge decreases both the values of ΔH^#^ and ΔS^#^. Molecular dynamics (MD) simulations suggested that the active-site arginine residue could adopt more rotamer conformations in the absence of the salt-bridge. The loss of this conformational freedom upon the formation of the transition state justified the observed reduction in the activation entropy. Finally, the implications on why thermophilic enzymes are less active at low temperatures are discussed.

## Results

### Removal of the Active-Site Salt-Bridge in Thermophilic PhAcP Reduces the Activation Energy of Catalysis

**Figure 2:**
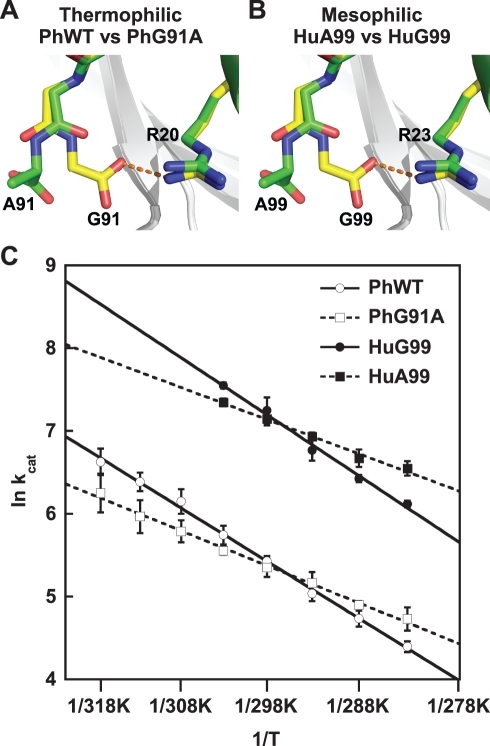
The salt-bridge restraining the active-site arginine residue resulted in a stronger temperature dependency of the acylphosphatase activity. (A) The active-site salt-bridge (orange dotted line) between the guanido group of Arg-20 and the C-terminal carboxyl group of Gly-91 in PhWT (in yellow) is removed in PhG91A (in green). (B) Replacing the C-terminal residue of HuAcP with a glycine residue facilitates the formation of the active-site salt-bridge (orange dotted line) in HuG99 (in yellow). Such salt-bridge is absent in the pseudo-wild-type HuA99 (in green). (C) The Arrhenius plots for PhWT (open circle), PhG91A (open square), HuG99 (filled circle), and HuA99 (filled square). The data showed that the salt-bridge bearing acylphosphatases (PhWT and HuG99, solid line) had a steeper slope than the variants (PhG91A and HuA99, dotted line) lacking the salt-bridge.

The salt-bridge between the active-site residue Arg-20 and the C-terminal carboxyl group is found only in thermophilic PhAcP but not in mesophilic HuAcP (Figure S1). To investigate how this salt-bridge affects the enzymatic activity, we need to engineer a variant of PhAcP in which the salt-bridge was disrupted without perturbing the active site. Molecular modeling suggested that the formation of the salt-bridge required the C-terminal residue Gly-91 to adopt an unusual phi angle of ∼180°. Thus, any non-glycine substitutions could disrupt the interaction. We then replaced the Gly-91 by an alanine residue to yield a variant of PhAcP (PhG91A) by site-directed mutagenesis. The substitution did not significantly affect the stability of the enzymes, for the apparent melting temperatures of PhWT (∼107°C) and PhG91A (∼106°C) were similar and there was no significant difference between the values of free energy of unfolding (ΔG_u_) for PhWT (58±7 kJ mol^−1^) and PhG91 (51±6 kJ mol^−1^) (Figure S2). The structure of PhG91A was determined by x-ray crystallography at 2.4 Å resolution (Table S1), and it is superimposable with the structure of wild-type PhAcP (PhWT) (C_α_ root-mean-square deviation (r.m.s.d.) of 0.21 Å between chain A of 1W2I [PhWT] and 2W4D [PhG91A]). Most importantly, the C-terminal alanine residue was confirmed to face away from the active-site arginine residue leading to the disruption of the salt-bridge (Figure 2A).

Next, the enzymatic activity of PhG91A and PhWT at different temperatures was determined and the results represented in the Arrhenius plot (ln *k*_cat_ versus 1/*T*). As shown in Figure 2C, PhWT and PhG91A both gave straight lines that intersected at ∼298 K. PhWT had a steeper slope denoting a stronger temperature dependency of the enzymatic activity than PhG91A. At ∼298 K, both enzymes shared similar *k_cat_* values (228±15 and 211±25 s^−1^ for PhWT and PhG91A, respectively) (Table 1). In terms of the activation energy (*E_a_*) of the reactions calculated from the slope of the Arrhenius plot, PhWT displayed a greater *E_a_* value (49.1±1.4 kJmol^−1^) than PhG91A (32.1±1.7 kJmol^−1^) (Table 1). Our data suggest that the removal of the salt-bridge between Arg20 and Gly91 results in a weaker temperature dependency of enzymatic activity. The consequences are that the enzymatic activity of PhWT will be higher at elevated temperatures but becomes more sluggish at lower temperatures. For example, at lower temperature, e.g. 283 K, PhG91A lacking the salt-bridge retains a significantly greater *k_cat_* value than PhWT (Figure 2C).

**Table 1:** Kinetics parameters of acylphosphatases with and without the active-site salt-bridge.

	Active-Site Salt-Bridge	k_cat_ (s^−1^)	E_a_ (kJ mol^−1^)	ΔG^#^ (kJmol^−1^)	ΔΔG^#^ (kJmol^−1^)	ΔH^#^ (kJmol^−1^)	ΔΔH^#^ (kJmol^−1^)	ΔS^#^ (J mol^−1^ K^−1^)	TΔS^#^ (kJmol^−1^)	TΔΔS^#^ (kJmol^−1^)
PhWT	Yes	228±15	49.1±1.4	59.5±0.02		46.6±1.4		−43±5	−12.9±1.4	
PhG91A	No	211±25	32.1±1.7	59.7±0.03	0.2±0.4	29.6±1.7	−17.0±2.2	−101±6	−30.1±1.7	−17.2±2.2
HuG99	Yes	1,405±225	52.5±2.5	55.0±0.04		50.0±2.5		−17±9	−5.0±2.5	
HuA99	No	1,268±100	29.5±1.9	55.3±0.02	0.3±0.4	27.0±1.9	−23.0±3.1	−95±6	−28.3±1.9	−23.3±3.1
HuWT (K99)	No	1,214±196	37.6±3.1	55.4±0.04	0.4±0.6	35.1±3.1	−14.9±4.0	−68±11	−20.2±3.1	−15.2±4.0

Values of the free energy (ΔG^#^), enthalpy (ΔH^#^), and entropy (ΔS^#^) of activation at 298 K were calculated by Δ*G* ^#^  =  *RT*(ln*k_B_T / h* − ln*k_cat_*), Δ*H* ^#^  =  *Ea* − *RT* , and TΔS^#^  =  ΔH^#^ − Δ*G* ^#^, where *k_B_* is the Boltzmann constant, *h* is the Planck constant, and *R* is the universal gas constant. ΔΔG^#^, ΔΔH^#^, and ΔΔS^#^ represent the changes of these thermodynamic parameters upon the removal of the salt-bridge between the active-site arginine residue and the C-terminal carboxyl group. For comparison, at 298 K, ΔG^#^, ΔH^#^, and TΔS^#^ of the uncatalyzed hydrolysis of benzoyl phosphate are 100.8, 102.2, and 1.4 kJ mol^−1^, respectively [12]. The K_m_ values were 0.10±0.03 mM for all acylphosphatases in temperature range studied.

### Introduction of the Active-Site Salt-Bridge into Mesophilic HuAcP Increases the Activation Energy of Catalysis

As shown earlier, the presence of the salt-bridge increases the activation energy of catalysis. We then questioned whether the introduction of the salt-bridge into the mesophilic homologue would result in the same observation. Two variants of HuAcP were constructed. Firstly, the C-terminal residue of HuAcP was substituted with a glycine residue (HuG99) to engage the salt-bridge with the active-site arginine residue. Secondly, a pseudo-wild-type HuAcP (HuA99) was created by replacing the C-terminal residue with an alanine residue to resemble PhG91A. Crystal structures of HuA99 and HuG99, resolved at 1.5 Å and 1.7 Å, respectively (Table S1), demonstrated that they were superimposable with a C_α_ r.m.s.d. value of 0.17 Å. In a good agreement with our original design, the salt-bridge between the C-terminal carboxyl group of Gly-99 and Arg-23 at the active site was only detected in HuG99, but not in the pseudo-wild-type HuA99 (Figure 2B).

Regarding the Arrhenius plot (Figure 2C), both HuG99 and HuA99 exhibited straight lines that intersected also roughly at 298 K. The salt-bridge bearing variant HuG99 illustrated a steeper slope of the Arrhenius plot, i.e. a stronger temperature dependency of the enzymatic activity, as compared to that of HuA99 lacking the salt-bridge. Importantly, the activation energy (52.5±2.5 kJmol^−1^) for HuG99 was analogous to that for PhWT (49.1±1.4 kJmol^−1^), while the activation energy for HuA99 (29.5±1.9 kJmol^−1^) and HuWT (37.6±3.1 kJmol^−1^) was comparable to that for PhG91A (32.1±1.7 kJmol^−1^) (Table 1). Taken together, our data suggest that the presence of the salt-bridge between the C-terminal carboxyl group and the active-site arginine residue increases the activation energy that results in a stronger temperature dependency of the enzymatic activity.

### Removal of the Active-Site Salt-Bridge Decreases the Entropy of Activation

**Figure 3:**
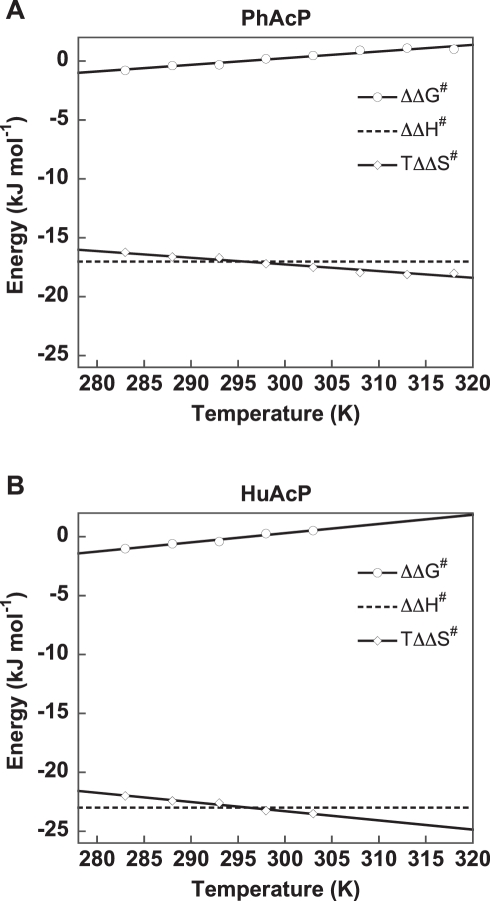
Removal of the active-site salt-bridge decreases both activation enthalpy and entropy. Changes in activation free energy (ΔΔG^#^, open circles, solid lines), activation enthalpy (ΔΔH^#^, dotted lines), and activation entropy (TΔΔS^#^, open diamond, solid lines) upon removal of the active-site salt-bridge were calculated as described in Table 1. As shown, removal of the salt-bridge leads to large negative values of both ΔΔH^#^ and TΔΔS^#^, while the effect on activation free energy at ∼298 K was minimal due to enthalpy-entropy compensation.

To provide further insights into how the active-site salt-bridge affected the enzymatic activity of acylphosphatases, we calculated and summarized the free energy (ΔG^#^), enthalpy (ΔH^#^), and entropy (ΔS^#^) of activation, as well as the changes of these parameters (ΔΔG^#^, ΔΔH^#^, ΔΔS^#^) upon the removal of the active-site salt-bridge (Table 1 and Figure 3). The values of ΔΔH^#^ and TΔΔS^#^ for HuWT were somewhat lower than those for HuA99. Nevertheless, the patterns of changes in the activation thermodynamics parameters were similar in both mesophilic and thermophilic acylphosphatases. Noteworthy, the removal of the active-site salt-bridges, i.e. PhWT versus PhG91A and HuG99 versus HuA99, led to large negative values of ΔΔS^#^ (Table 1).

As shown in Table 1 and Figure 3, the large negative values of TΔΔS^#^ due to the removal of the salt-bridge were accompanied by large negative values of ΔΔH^#^. At ∼298 K, these two effects canceled out each other, and therefore, the changes in ΔG^#^ and the resulting reaction rates were minimal (Table 1). At <298 K, the enthalpic term (ΔΔH^#^) was smaller than the entropic term (TΔΔS^#^). As a result, acylphosphatases without the salt-bridge (PhG91A, HuA99) were more active than those with the salt-bridge (PhWT, HuG99) (Figures 2 and 3). Conversely, at >298 K, the acylphosphatases with the salt-bridge became more active (Figures 2 and 3).

### Removal of the Active-Site Salt-Bridge Decreases the Entropy of Binding

To investigate if the active-site salt-bridge affects substrate binding, we have performed isothermal titration calorimetry to measure the thermodynamics parameters of substrate binding for variants of acylphosphatases using a substrate analogue, S-benzyloxycarbonyl-thiosulfonate (Table 2 and Figure S3). Our results showed that the removal of the active-site salt-bridge had minimal effect on the substrate binding affinity (i.e. similar values of K_a_ and ΔG_b_) but resulted in significant decreases in both enthalpy and entropy of binding (Table 2). The entropic contribution (TΔΔS_b_) of removing the active-site salt-bridge to substrate binding was −6.0 and −3.8 kJ mol^−1^ for thermophilic and mesophilic AcP, respectively. Noteworthy, these values were much less negative than the corresponding changes in activation entropy (TΔΔS^#^, Table 1), which were −17.2 and −23.3 kJ mol^−1^, suggesting that upon removal of the active-site salt-bridge, the system loses more entropy in the formation of the transition state than the formation of the enzyme-substrate complex.

**Table 2:** Thermodynamics parameters for binding of substrate-analogue determined by isothermal titration calorimetry.

	Active-Site Salt-Bridge	K_a_ (10^3^ M^−1^)	ΔG_b_ (kJ mol^−1^)	ΔΔG_b_ (kJ mol^−1^)	ΔH_b_ (kJ mol^−1^)	ΔΔH_b_ (kJ mol^−1^)	ΔS_b_ (J mol^−1^ K^−1^)	TΔS_b_ (kJ mol^−1^)	TΔΔS_b_ (kJ mol^−1^)
PhWT	Yes	6.6±0.4	−21.8±0.2		−6.7±0.4		50±1	15.1±0.4	—
PhG91A	No	7.5±0.4	−22.1±0.1	−0.3±0.2	−13.1±1.0	−6.4±1.1	30±3	9.0±1.0	−6.1±1.1
HuG99	Yes	5.4±0.2	−21.3±0.5		−7.0±0.4		48±1	14.3±0.6	—
HuA99	No	5.0±0.6	−21.1±0.1	0.2±0.5	−10.6±0.8	−3.6±0.9	35±3	10.5±0.8	−3.8±1.0
HuWT (K99)	No	5.1±0.1	−21.1±0.5	0.2±0.7	−12.1±3.4	−5.1±3.4	30±13	9.0±3.5	−5.3±3.6

Substrate-analogue, S-benzyloxycarbonyl-thiosulfonate, was titrated to the variants of acylphosphatases to obtain values of association constant (K_a_) and enthalpy (ΔH_b_) of binding at 298 K. Free energy (ΔG_b_) and entropy (ΔS_b_) of binding were calculated by: Δ*G_b_*  =  −*RT*ln*K_a_* and *T*Δ*S_b_*  =  Δ*H_b_* − Δ*G_b_*, where *R* is the universal gas constant. ΔΔG_b_, ΔΔH_b_, and ΔΔS_b_ represent the changes of these thermodynamic parameters upon the removal of the salt-bridge between the active-site arginine residue and the C-terminal carboxyl group.

### MD Simulations Showed that the Active-Site Arginine Residue Populates More Rotamer Conformations after the Breakage of the Active-Site Salt-Bridge

We have shown that the removal of the active-site salt-bridge decreased the activation entropy, suggesting an increase in the local flexibility at the active site. Next, we performed the MD simulations to further characterize the local flexibility of the active site affected by the salt-bridge. Three 10 ns trajectories were obtained for each of the acylphosphatases studied, namely PhWT, PhG91A, HuG99, and HuA99. The MD simulations were stable, with values of C_α_ root-mean-square deviation below ∼1.5 Å (Figure S4). The distance between the C-terminal carboxyl group and the guanido group of the active-site arginine residue was less than 4 Å throughout the entire simulation of PhWT and HuG99, denoting the presence of the salt-bridges in these proteins. On the other hand, the distance was ∼6 Å throughout the simulation of PhG91A and HuA99, suggesting the salt-bridge was broken. Moreover, for PhG91A and HuA99, the removal of the salt-bridge did not affect backbone flexibility significantly, as indicated by the comparable values of C_α_ root-mean-square fluctuations derived from the MD simulations (Figure S5).

**Figure 4:**
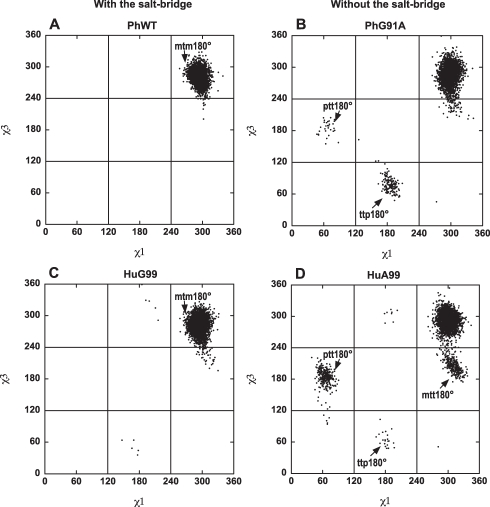
The active-site salt-bridge restricts the side-chain conformational freedom of the active-site arginine residue. The local flexibility of the active-site arginine residue (Arg-20 in PhWT and PhG91A or Arg-23 in HuG99 and HuA99) was examined by MD simulations. For acylphosphatases with the salt-bridge (PhWT and HuG99), the χ1, χ2, χ3, and χ4 dihedral angles of the arginine residue were confined to the values of ∼300°, ∼180°, ∼300°, and ∼180°, respectively. In other words, the side-chain of the arginine residue populates mainly in the mtm180° rotamer. According to the convention of Lovell et al. [18], “p,” “t,” and “m” refers to dihedral angles of 60°, 180°, and 300°, respectively. For acylphosphatases without the salt-bridge (PhG91A and HuA99), transitions from the mtm180° to other rotamer conformations (ptt180°, ttp180°, and mtt180°) were evident.

The notable change in the flexibility upon the removal of the active-site salt-bridge was localized in the side-chain conformation of the active-site arginine residue (Arg-20 in PhAcP and Arg-23 in HuAcP). In the crystal structure of acylphosphatases, the active-site arginine residue adopted the mtm180° rotamer conformation (named after the convention of Lovell et. al., 2000 [18]) (Figure 4). In the simulations of the salt-bridge bearing PhWT and HuG99, the side-chains of the arginine residues populated mainly the native mtm180° rotamer conformation (Figure 4A and C). In contrast, despite the mtm180° rotamer conformation, transitions to ptt180°, ttp180°, and mtt180° rotamers were prominent in the MD trajectories of PhG91A and HuA99 (Figure 4B and D). Our results suggest that the removal of the active-site salt-bridge allows the arginine residue to populate several more rotamer conformations other than the orientated mtm180° rotamer conformation in the salt-bridge structure.

## Discussion

**Figure 5:**
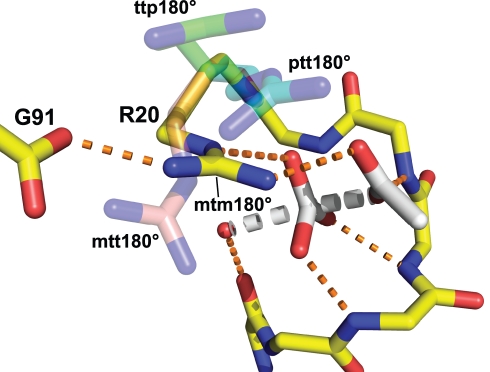
The active-site arginine residue of acylphosphatase adopts the mtm180° rotamer conformation for catalysis. The model of enzyme-transition-state complex was derived from the model of enzyme-substrate complex [14] by orientating the phosphorus atom towards the water molecule. Hydrogen bonds and salt-bridges are denoted by orange dotted lines. According to the proposed model, the guanido group of Arg-20 can form charge-charge interactions to stabilize the transition-state when the residue adopts the mtm180° rotamer conformation. For the mtt180°, ttp180°, and ptt180° rotamer conformations, the guanido group is too far away to form any favorable interactions with the transition state.

In this study, we used a “mirror-image” mutation approach [19] to investigate the role of the salt-bridge that restricts the flexibility of the active-site arginine residue on the enzymatic activity of acylphosphatases. Our data clearly demonstrated that the removal of the active-site salt-bridge in the thermophilic PhAcP decreased both the ΔH^#^ and ΔS^#^, while a parallel trend was observed when the salt-bridge was introduced in the mesophilic HuAcP. Our results strongly indicate that the salt-bridge increases the activation entropy by rigidifying the active-site arginine residue. From the MD simulation analysis, in the absence of the salt-bridge, the active-site arginine residue populates a broader distribution of conformations in the ground state (Figure 4). That TΔΔS^#^ took more negative values than TΔΔS_b_ (Tables 1 and 2) suggests that the majority of these degrees of freedom of the arginine residue are lost upon the formation of the transition state, in which a highly restrained positioning of the active-site residue is required for the catalysis to optimally occur. Indeed, based on our previously proposed model of an enzyme-substrate complex of the acylphosphatase [14], the active-site arginine residue has to adopt the mtm180° rotamer conformation in order to stabilize the negative charges developed on the carbonyl group of the leaving group during the formation of the transition state, while other rotamer conformations are not productive (Figure 5). This loss of the conformational freedom results in a more negative value of ΔS^#^ as in the cases of PhG91A and HuA99. In our case, the active-site salt-bridge contributes to a decrease of ∼20 kJ mol^−1^ in the entropic penalty at 298 K (Table 1), which can translate into a >3,000-fold increase in *k_cat_* if the ΔH^#^ remains constant. From the entropic point of view, rigidifying the active-site residue should increase the enzymatic activity rather than decrease it.

The reduced activity of thermophilic acylphosphatase at low temperatures is caused by the accompanying increases in activation enthalpy that counteracts the entropic term (Table 1). The cause of such enthalpy-entropy compensation is intriguing. It has been argued that flexibility can contribute to the lowering of activation barriers because it facilitates sampling of conformational sub-states that have lower barriers for catalysis to occur [20],[21]. In another view by Warshel and co-workers, enzyme catalysis is determined mainly by electrostatic reorganization energy [22], and the reduction of ΔH^#^ in mesophilic and psychrophilic enzymes is probably a result of the reduction of the reorganization energy but not changes in flexibility [23]. Another possibility is that the presence of a nearby negatively charged C-terminal carboxyl group may destabilize the transition state and thereby increase the activation enthalpy. Nevertheless, the enthalpy-entropy compensation appears to be a general property of weak intermolecular interactions. According to a simple model by Dunitz, the enthalpic and entropic contributions to the free energy for weak intermolecular interactions should compensate each other at ∼300 K [24].

Why is the active-site salt-bridge present in thermophilic acylphosphatases but absent in mesophilic homologs? Our results suggest that the active-site salt-bridge increases the enzymatic activity at higher temperatures where the entropic term dominates (Figure 2). For example, the k_cat_ value for PhWT (760 s^−1^) at 318 K was significantly higher than that for PhG91A (520 s^−1^). From this point of view, rigidifying the active-site residue is a structural adaption of thermophilic acylphosphatases that favors enzymatic activity at high temperatures. Due to enthalpy-entropy compensation, the salt-bridge also leads to a stronger temperature dependency in enzymatic activity so that the thermophilic acylphosphatase becomes less active at low temperatures. In fact, the low-temperature activities (at <298 K) of the thermophilic acylphosphatase (like in the case of PhG91A) can be improved by the removal of the salt-bridge that lowers the activation enthalpy and increases the local flexibility of the active-site arginine residues. This observation is consistent with the suggestion that psychrophilic enzymes adapt to remain active at low temperatures by lowering the activation enthalpy [8],[9],[25],[26].

Our results showed that the contribution of the active-site salt-bridge to the thermal stability of the acylphosphatase, if any, is small (Figure S2). Although many sequence-structure comparisons suggest that thermophilic proteins tend to have more salt-bridges than their mesophilic homologues [27]–[29], whether salt-bridges stabilizes proteins is context-dependent because the favorable electrostatic interaction of opposite charges may be offset by dehydration penalty and the entropic cost of fixing the salt-bridging groups [30]–[33]. Moreover, surface-charged residues may stabilize proteins through long-range electrostatic interactions [34]–[39]. Using a genetic algorithm that optimizes the surface electrostatic interactions, Makhatadze and co-workers introduced five substitutions to human acylphosphatase and improved its melting temperature by ∼10°C without affecting its enzymatic activity [40]. Their results suggest that one can improve the thermal stability of an enzyme without compromising its activity.

Noteworthy, our data also suggest that the active-site salt-bridge is not the sole factor contributing to the reduced activity of the thermophilic acylphosphatase at low temperatures. For instance, the thermophilic PhG91A is consistently less active than the mesophilic HuA99 although both enzymes lack the active-site salt-bridge (Figure 2C). While the two enzymes have similar values of activation enthalpy (i.e. similar slope in the Arrhenius plot), the differences in enzymatic activity are a result of PhG91A having a more negative value of activation entropy (Table 1). As all active-site residues in acylphosphatases are highly conserved, our data support the conclusion that substitutions at non-active-site residues play a critical role in decreasing the activation entropy and hence the enzymatic activity of the thermophilic acylphosphatase. That non-active-site residues do affect enzymatic activity has also been demonstrated in other enzymes [41],[42]. Although how non-active-site substitutions affect activation entropy is not known, the reduction in activation entropy is unlikely to be caused by rigidifying the active site of the thermophilic enzyme, which is supposed to increase the activation entropy rather than decrease it. Apparently, substitutions that increase the activation entropy without affecting activation enthalpy may be a good strategy to improve the enzymatic activity of thermophilic enzymes at low temperatures.

## Materials and Methods

### Construction of Mutants

The fragments of mutants were amplified by polymerase chain reaction method and subcloned into pET507a, an in-house modified vector with a multiple cloning site inserted between the *Nco*I and *BamH*I sites of pET3d (Novagen). DNA sequencing was performed to check the sequence of all mutants created. The primers used for the mutations were as follows: PhG91A forward (5′ TAACTACCATGGCCATAGTTAGGGCTCAC 3′) and reverse (5′ TAACTAGGATCCTCACGCAACGATCCTGAA 3′); pseudo-WT HuA99 forward (5′ TAACTACCATGGCAGAAGGAAACACCCTG 3′); reverse (5′ TAGCGCGGATCCTTACGCTACAATTTGGAAG 3′); and HuG99 reverse (5′ TAGCGCGGATCCTTAGCCTACAATTTGGAAG 3′).

### Protein Samples Preparation

Protein samples of all acylphosphatases and their mutants were expressed and purified as described previously [14],[16].

### Enzymatic Assay for Acylphosphatase

The continuous optical enzymatic assay for acylphosphatase was performed as previously described [14] using benzoyl phosphate as substrate. The assay was performed in triplicates by incubating substrate from 0.05 to 2.0 mM with acylphosphatases from 0.8 to 1.5 nM in 0.1 M sodium acetate buffers at pH 5.3. The rate of hydrolysis was monitored by the decrease of the absorbance at 283 nm. For the mesophilic acylphosphatases, the assay was performed at 283, 288, 293, 298, and 303 K. For the thermophilic acylphosphatases, the temperature range was extended to include 308, 313, and 318 K. Enzyme kinetics parameters were obtained up to 318 K because the substrate benzoyl phosphate became too labile at higher temperatures.

### Isothermal Titration Calorimetry

Calorimetric measurements were carried out using a Nano ITC isothermal calorimeter (TA Instruments) at 298 K. Substrate analogue S-benzyloxycarbonyl-thiosulfonate (30 mM) (Sigma-aldrich) was titrated in 25 injections of 4 µl each to the protein sample (1.5 mM) in 0.1 M sodium acetate buffer at pH 5.3 in a 1 ml sample cell. The data were analyzed by the program NanoAnalyze provided by the manufacturer.

### Structure Determination

Crystals of PhG91A, HuA99, and HuG99 were grown using the sitting-drop-vapor diffusion method at 289 K. The crystallization conditions are summarized in Table S1. The crystals were cryoprotected by soaking in 25% (w/v) glycerol for PhG91A or polyethylene glycol-400 (PEG400) for HuA99 and HuG99 in their corresponding mother liquors. The crystals were then loop-mounted and flash-cooled in liquid nitrogen. X-ray diffraction datasets were collected at 100 K using an in-house R-AXIS IV ++ imaging-plate system and a rotating copper-anode x-ray source (Rigaku MicroMax-007 with VariMax optics). The diffraction data were processed with MOSFLM, SCALA, and TRUNCATE in the CCP4 suite [43]. The structures were resolved by the molecular replacement using the crystal structures of wild-type PhAcP [14] and HuAcP [16] as the search templates. Models were built by XTALVIEW [44] and refined by the programs CNS [45] and REFMAC5 [43]. The refined structures were validated by PROCHECK [46] and WHATIF [47]. The Ramachandran analysis was performed using the program MOLPROBITY [48].

### Molecular Dynamic (MD) Simulations

The details of the MD simulations are described in Text S1. In brief, all simulations were performed using GROMACS version 3.3 with the all-atom OPLSAA force field [49], using a 0.002 ps time step for 10 ns. Three MD trajectories were obtained for each of the PhWT, PhG91A, HuG99, and HuA99, and the structures were analyzed at every 1 ps interval.

## Supporting Information

Figure S1 The salt-bridge between the active-site arginine residue and the C-terminal carboxylate group is only found in thermophilic AcPs but not in mesophilic AcPs. (A) Superimposition of crystal structures of thermophilic (left panel) and mesophilic (right panel) acylphosphatases from *P. horikoshii* (PhWT, green), *T. thermophilus* (pink), *S. solfataricus* (magenta), human (white), cattle (cyan), Drosophila (blue), and *B. subtilis* (yellow). (B) Sequence alignment of the C-terminal residues of AcP. The C-terminal carboxylate group is located at the end of strand 5 (β5). In PhWT, the formation of the salt-bridge is facilitated by having a glycine at the C-terminus, which can adopt an unusual φ angle of ∼180°. The salt-bridge is found in both chain A and chain B of PhWT (PDB code: 1W2I). The electron density of the Gly-91 in chain B is weaker, suggesting an increase of disorder of the residue in chain B. In the case of AcPs from *T. thermophilus* and *S. solfatericus*, the C-terminal carboxylate groups are brought in the position to form the salt-bridge by having one less residue at the C-termini.
(TIF)

Figure S2 The contribution of the active-site salt-bridge to the thermal stability of acylphosphatases is minimal. (A) The thermal unfolding was monitored by differential scanning calorimetry. The apparent melting temperatures estimated for the irreversible thermal unfolding of PhWT and PhG91A were ca. 107°C and 106°C, respectively. (B) The free energy of unfolding was determined by guanidine-induced denaturation at 25°C. The ΔG_u_, mid-point of transition and m-values were 58±7 kJ mol^−1^, 5.30±0.04 M, and 10.9±1.2 for PhWT, and 51±6 kJ mol^−1^, 5.23±0.05 M, and 9.7±1.1 for PhG91A.
(TIF)

Figure S3 Isothermal titration calorimetry. S-benzyloxycarbonyl-thiosulfonate at 30 mM was titrated in 25 injections of 4 µl each to 1.5 mM protein samples of PhWT, PhG91A, HuG99, HuA99, and HuWT in a 1 ml sample cell. The data were fitted to a single site model to obtain values of association constant (K_a_) and enthalpy of binding (ΔH_b_).
(TIF)

Figure S4 C_α_ root-mean-square deviation from the starting structure as a function of time. Three 10 ns MD trajectories were run at 298 K for (A) PhWT, (B) PhG91A, (C) HuG99, and (D) HuA99.
(TIF)

Figure S5 Cα root-mean-square fluctuations (r.m.s.f.) derived from the ensembles of MD-generated structures. As shown, removal of the active-site salt-bridge does not affect significantly the values of Cα r.m.s.f. in (A) thermophilic acylphosphatases and (B) human acylphosphatases.
(TIF)

Table S1 Structure determination of acylphosphatase variants—crystallization, data collection, and refinement statistics.
(PDF)

Text S1 Supporting methods. Supporting information describes the detailed procedures for the determination of free energy of unfolding, differential scanning calorimetry, and molecular dynamics simulation.
(DOC)

## Abbreviations

ΔG^#^: ΔH^#^, ΔS^#^: free energy, enthalpy, entropy of activation
ΔG_b_: ΔH_b,_ ΔS_b_: free energy, enthalpy, entropy of substrate-analogue binding
AcP: acylphosphatase
CD: circular dichroism
Hu: human
MD: molecular dynamics
Ph: *Pyrococcus horikoshii*
r.m.s.d.: root mean square deviation
WT: wild-type
