# Supplementary figures and images for: A Rigidifying Salt-Bridge Favors the Activity of Thermophilic Enzyme at High Temperatures at the Expense of Low-Temperature Activity
Source: PLoS Biol. 2011 Mar 15;9(3):e1001027. doi: 10.1371/journal.pbio.1001027 (PMC3057955; doi:10.1371/journal.pbio.1001027)

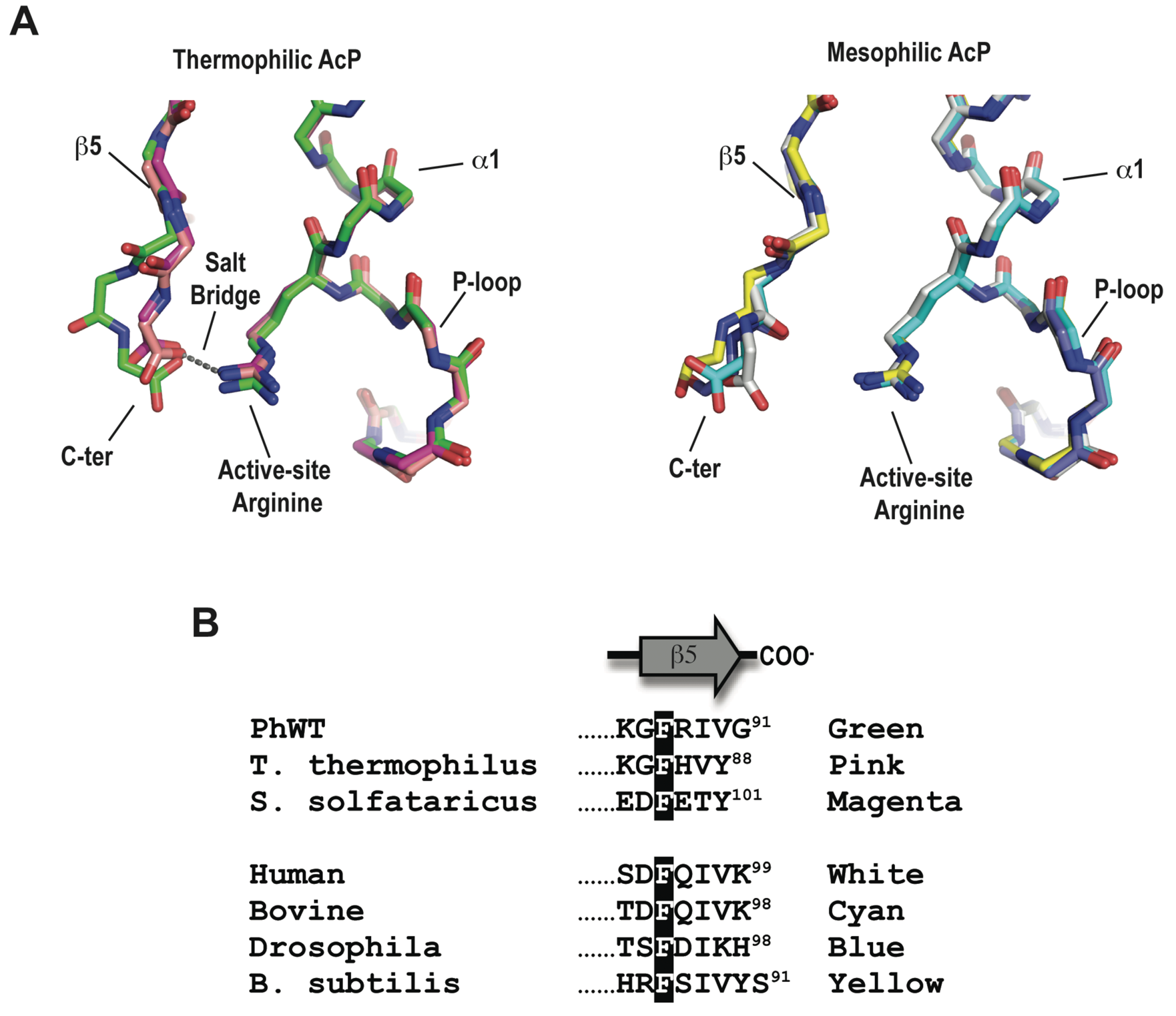

Supplement: Figure S1 — The salt-bridge between the active-site arginine residue and the C-terminal carboxylate group is only found in thermophilic AcPs but not in mesophilic AcPs. (A) Superimposition of crystal structures of thermophilic (left panel) and mesophilic (right panel) acylphosphatases from P. horikoshii (PhWT, green), T. thermophilus (pink), S. solfataricus (magenta), human (white), cattle (cyan), Drosophila (blue), and B. subtilis (yellow). (B) Sequence alignment of the C-terminal residues of AcP. The C-terminal carboxylate group is located at the end of strand 5 (β5). In PhWT, the formation of the salt-bridge is facilitated by having a glycine at the C-terminus, which can adopt an unusual φ angle of ∼180°. The salt-bridge is found in both chain A and chain B of PhWT (PDB code: 1W2I). The electron density of the Gly-91 in chain B is weaker, suggesting an increase of disorder of the residue in chain B. In the case of AcPs from T. thermophilus and S. solfatericus, the C-terminal carboxylate groups are brought in the position to form the salt-bridge by having one less residue at the C-termini. (TIF) [file pbio.1001027.s001.tif]

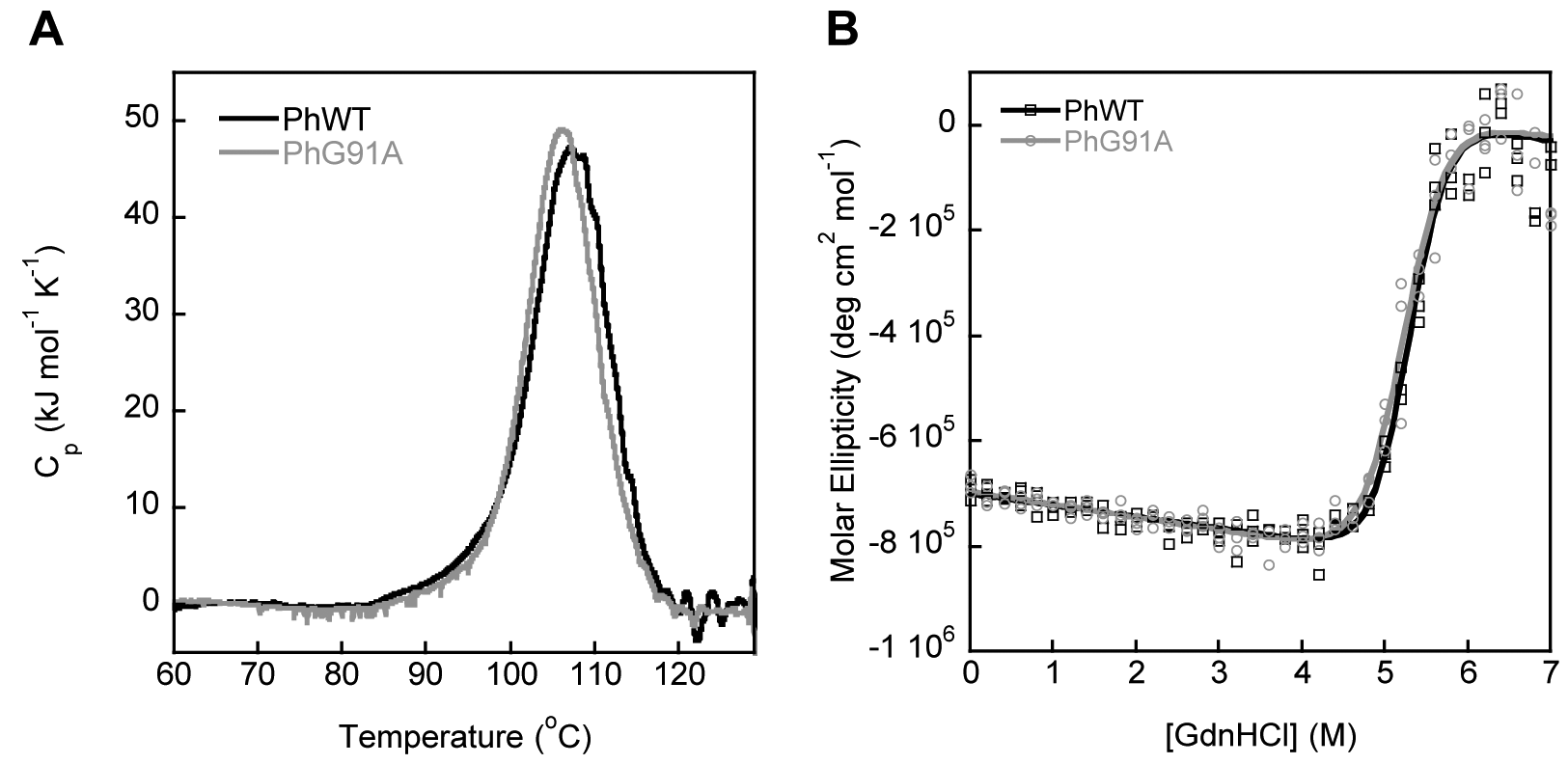

Supplement: Figure S2 — The contribution of the active-site salt-bridge to the thermal stability of acylphosphatases is minimal. (A) The thermal unfolding was monitored by differential scanning calorimetry. The apparent melting temperatures estimated for the irreversible thermal unfolding of PhWT and PhG91A were ca. 107°C and 106°C, respectively. (B) The free energy of unfolding was determined by guanidine-induced denaturation at 25°C. The ΔGu, mid-point of transition and m-values were 58±7 kJ mol−1, 5.30±0.04 M, and 10.9±1.2 for PhWT, and 51±6 kJ mol−1, 5.23±0.05 M, and 9.7±1.1 for PhG91A. (TIF) [file pbio.1001027.s002.tif]

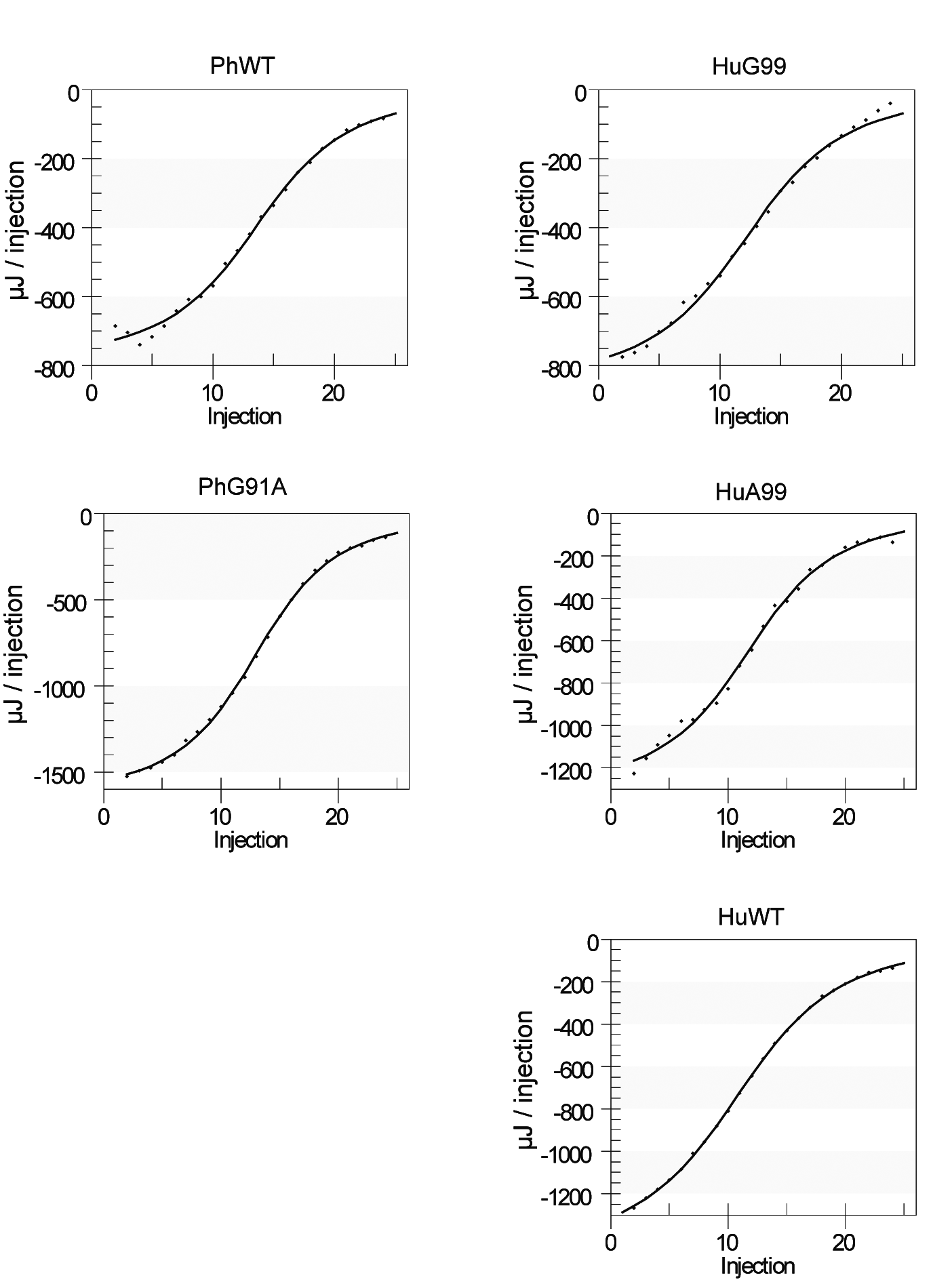

Supplement: Figure S3 — Isothermal titration calorimetry. S-benzyloxycarbonyl-thiosulfonate at 30 mM was titrated in 25 injections of 4 µl each to 1.5 mM protein samples of PhWT, PhG91A, HuG99, HuA99, and HuWT in a 1 ml sample cell. The data were fitted to a single site model to obtain values of association constant (Ka) and enthalpy of binding (ΔHb). (TIF) [file pbio.1001027.s003.tif]

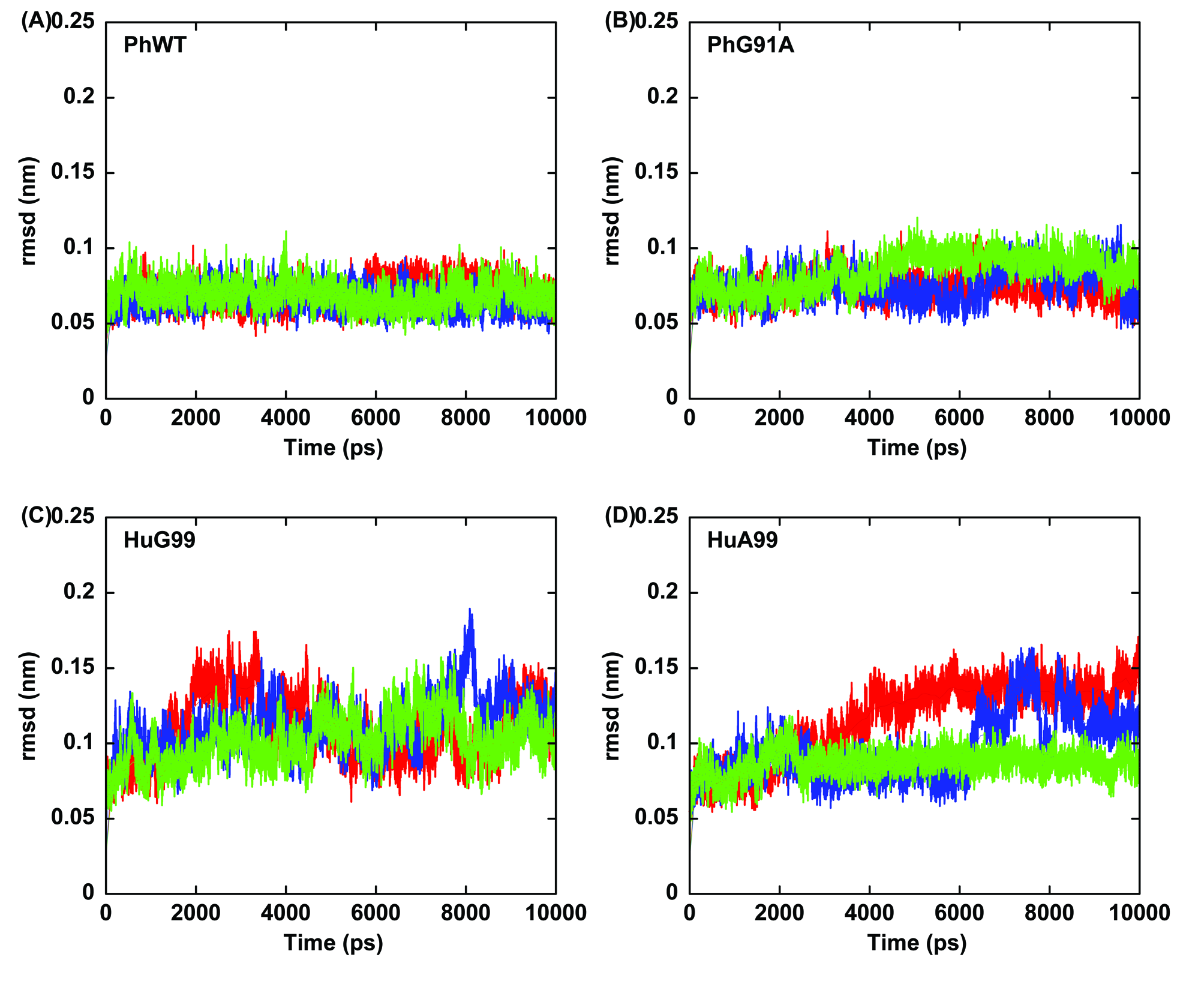

Supplement: Figure S4 — Cα root-mean-square deviation from the starting structure as a function of time. Three 10 ns MD trajectories were run at 298 K for (A) PhWT, (B) PhG91A, (C) HuG99, and (D) HuA99. (TIF) [file pbio.1001027.s004.tif]

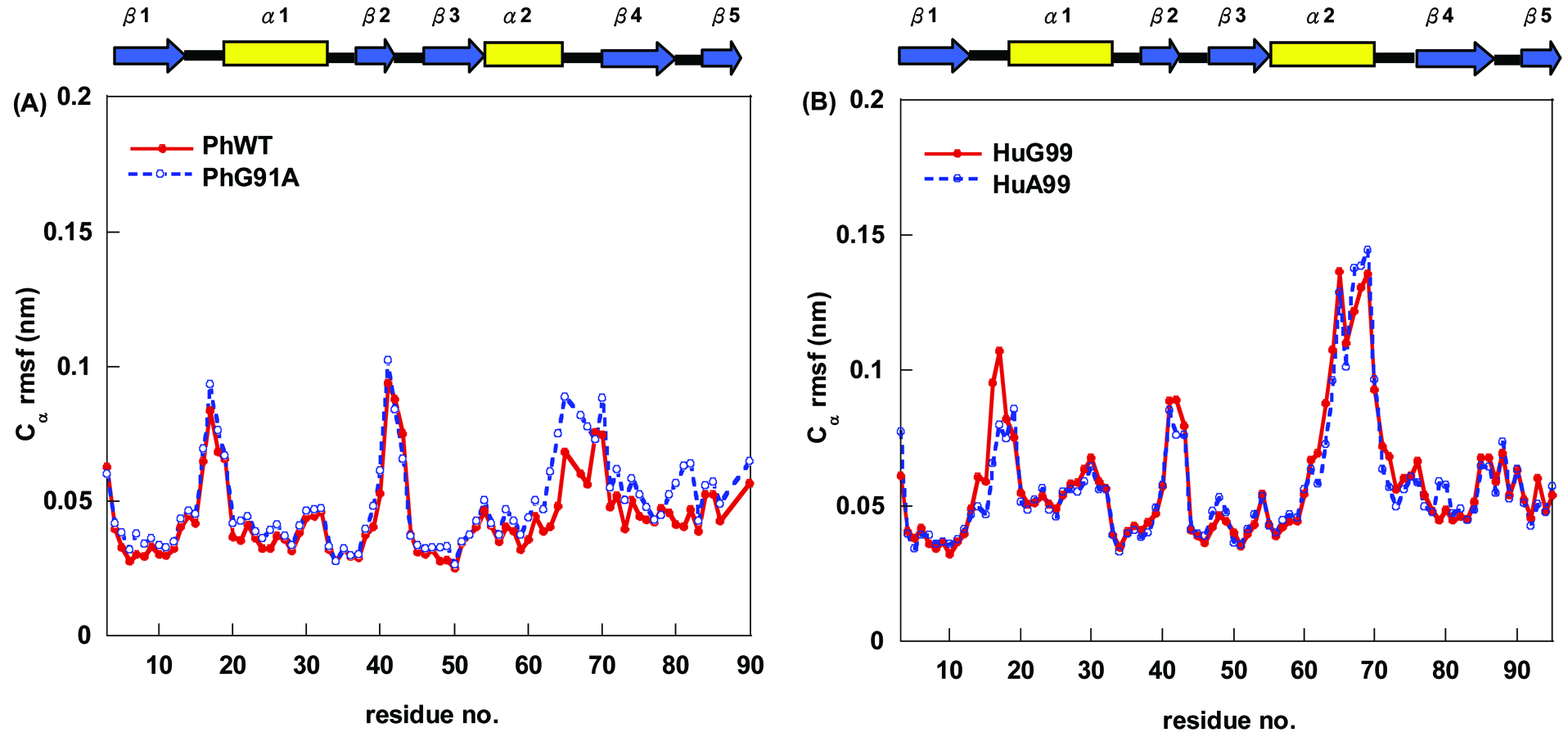

Supplement: Figure S5 — Cα root-mean-square fluctuations (r.m.s.f.) derived from the ensembles of MD-generated structures. As shown, removal of the active-site salt-bridge does not affect significantly the values of Cα r.m.s.f. in (A) thermophilic acylphosphatases and (B) human acylphosphatases. (TIF) [file pbio.1001027.s005.tif]
