# Supplementary material for: A Rigidifying Salt-Bridge Favors the Activity of Thermophilic Enzyme at High Temperatures at the Expense of Low-Temperature Activity
Source: PLoS Biol. 2011 Mar 15;9(3):e1001027. doi: 10.1371/journal.pbio.1001027 (PMC3057955; doi:10.1371/journal.pbio.1001027)
Supplement: Table S1 — Structure determination of acylphosphatase variants—crystallization, data collection, and refinement statistics. (PDF) [file pbio.1001027.s006.pdf]

Table S1. Structure determination of acylphosphatase variants - crystallization, data collection and refinement statistics.

	PhG91A	HuA99	HuG99
Crystallization conditions	0.6M Na/K Phosphate, pH8.5, 25% glycerol	0.1M Bis-Tris, 0.1M Sodium acetate, pH6.0, 25% PEG3350	2.4M Sodium malonate, pH7.0
**Diffraction data collection (Values in parenthesis are for the highest-resolution shell)**			
X- ray source	Cu K α	Cu K α	Cu K α
Space group	$P2_12_12$	$P2_12_12_1$	$P2_12_12_1$
Resolution (Å)	44.18- 2.40 (2.49-2.40)	14.31- 1.52 (1.60-1.52)	41.03- 1.70 (1.79-1.70)
Unit cell dimension (Å)	a=46.046; b=87.39; c= 153.60	a=24.604; b=57.861; c=59.095	a=24.206; b=57.339; c=58.777
Multiplicity	2.92 (2.74)	6.7 (6.5)	6.5 (6.3)
Completeness (%)	98.9 (97.5)	99.9 (100.1)	99.9 (99.5)
Mean $I/\sigma(I)$	10.7 (4.2)	30.7 (9.2)	21.3 (4.4)
R_{merge} [a]	0.070 (0.199)	0.039 (0.184)	0.078 (0.338)
Unique reflections	24753	9045	12894
**Structural Refinement**			
R_{cryst}/R_{free} (%) [b]	22.7/ 25.2	19.6/ 22.8	18.7/ 24.1
Model			
No. of protein molecules	6	1	1
Protein atoms	4308	765	768
Heterogen atoms	41	0	0
Water atoms	125	107	85
RMS deviation from ideal values			
Bond distance (Å)	0.007	0.012	0.016
Bond angles (deg.)	1.50	1.35	1.55
Ramachandran analysis using MOLPROBITY			
Favored region (%)	99.8	97.8	98.9
Other allowed region (%)	0.2	2.2	1.1
Outlier (%)	0	0	0

[a] $R_{merge} = \sum_{hkl}\sum_{n}\left|I(hkl)_n - \langle I(hkl)_n\rangle\right| / \sum_{hkl}\sum_{n} I(hkl)_n$

[b] $R_{cryt} = \sum\left\|F_o\right| - \left|F_c\right\| / \sum\left|F_o\right|$, where $|F_o|$ and $|F_c|$ are the observed and calculated amplitudes, respectively. The free set contains 5% of the total reflections.
