# Supplementary material for: A Rigidifying Salt-Bridge Favors the Activity of Thermophilic Enzyme at High Temperatures at the Expense of Low-Temperature Activity
Source: PLoS Biol. 2011 Mar 15;9(3):e1001027. doi: 10.1371/journal.pbio.1001027 (PMC3057955; doi:10.1371/journal.pbio.1001027)
Supplement: Text S1 — Supporting methods. Supporting information describes the detailed procedures for the determination of free energy of unfolding, differential scanning calorimetry, and molecular dynamics simulation. (DOC) [file pbio.1001027.s007.doc]

**Supporting Methods**

**Determination of free energy of unfolding by guanidine-induced denaturation**

Protein samples of 25 μM were equilibrated in 0 - 7.2 M guanidine hydrochloride (GdnHCl) with 10 mM sodium acetate buffer, pH 5.3 for 30 min at 25 ºC. The concentration of GdnHCl was determined by refractive index measurements [1] using a Leica AR200 refractometer. Ellipticity at 222 nm was measured at 25ºC using a 1-mm path length cuvette with a JASCO J810 spectropolarimeter equipped with a peltier type temperature controller. The data were fitted by non-linear regression to a two-state model [2] using: y_obs_ = { (a_n_ + b_n_ [D]) + (a_u_ + b_u_ [D] ) e^-ΔG(D)/RT^ } / ( 1 + e^-ΔG(D)/RT^), where y_obs_ is the observed molar ellipticity at 222 nm; a_n_ and b_n_ are the y-intercept and slope of the pre-transition baseline; a_u_ and b_u_ are the y-intercept and slope of the post-transition baseline; R is the gas constant; T is the temperature in Kelvin; [D] is the concentration of denaturant; ΔG_(D)_ is the free energy of unfolding at [D]. The free energy of unfolding, ΔG_u_, was obtained by the linear extrapolation model [2]: ΔG_u_ = m [D]_1/2_ , where m is the m-value, and [D]_1/2_ is the concentration of denaturant at the mid-point of transition. The mean and standard deviation of three independent measurements were reported.

**Differential scanning calorimetry**

Measurements were carried out using the Nano III Differential Scanning Calorimeter (TA instruments). Buffer solutions and protein samples of concentration 1 mg/mL in 10 mM sodium acetate buffer, pH 5.3 were thoroughly degassed before being loaded into cells. Baseline was obtained by filling both sample and reference cells with buffer. Temperatures were scanned at a rate of 1 °C/min from 40 – 130 °C for thermophilic acylphosphatases. The absence of heat releasing peak upon reverse scanning indicated that thermal unfolding of PhWT and PhG91A was irreversible. The apparent melting temperatures were estimated by the mid-point of transition.

**Molecular dynamic (MD) simulations**

All simulations were performed using GROMACS version 3.3 with the all-atom OPLSAA force field [3]. The starting structures were derived from the crystal structures of PhWT, PhG91A, HuG99, and HuA99 (PDB codes: 1W2I, 2W4D, 2W4P, and 2W4C, respectively), and were solvated in dodecahedron TIP4P water box with walls at least 1.0 nm away from any protein atoms. The ionization of the charged residues was set to mimic a low-neutral pH environment (Lys, Arg, and His were protonated; Asp and Glu were ionized). The system was first minimized for 500 cycles of the steepest descent. Water molecules were then relaxed in 100-ps MD simulation in NPT ensemble with positional restraints on the protein atoms. For each protein, three 10-ns MD simulations were run, with different random seeds to generate starting velocities from a Maxwellian distribution at 298 K. With periodic boundary conditions, the size of the box was kept constant throughout the simulation. Lennard-Jones interactions were calculated with a 1.4 nm cut-off, and the Particle-Mesh-Ewald summation was used to calculate the long-range electrostatic interactions. Bond lengths were then constrained using the LINCS algorithm [4]. MD simulation was performed using a 0.002-ps time step, and the structures were analyzed at every 1-ps interval.

**Reference**

1. Pace CN (1986) Determination and analysis of urea and guanidine hydrochloride denaturation curves. Methods Enzymol 131: 266-280.

2. Santoro MM, Bolen DW (1988) Unfolding free energy changes determined by the linear extrapolation method. 1. Unfolding of phenylmethanesulfonyl alpha-chymotrypsin using different denaturants. Biochemistry 27: 8063-8068.

3. Lindahl E, Hess B, van der Spoel D (2001) GROMACS 3.0: A package for molecular simulation and trajectory analysis. J Mol Mod 7: 306-317.

4. Hess B, Bekker H, Berendsen HJC, Fraaije JGEM (1997) LINCS: A linear constraint solver for molecular simulations. J Comp Chem 18: 1463-1472.
